# Role of the Plasma Membrane Transporter of Organic Cations OCT1 and Its Genetic Variants in Modern Liver Pharmacology

**DOI:** 10.1155/2013/692071

**Published:** 2013-07-31

**Authors:** Elisa Lozano, Elisa Herraez, Oscar Briz, Virginia S. Robledo, Jorge Hernandez-Iglesias, Ana Gonzalez-Hernandez, Jose J. G. Marin

**Affiliations:** ^1^Laboratory of Experimental Hepatology and Drug Targeting (HEVEFARM), Biomedical Research Institute of Salamanca (IBSAL), University of Salamanca, Campus Miguel de Unamuno, E.D. S-09, 37007 Salamanca, Spain; ^2^Department of Physiology and Pharmacology, University of Salamanca, Campus Miguel de Unamuno, E.D. S-09, 37007 Salamanca, Spain; ^3^National Institute for the Study of Liver and Gastrointestinal Diseases (CIBERehd), Instituto de Salud Carlos III, Sinesio Delgado 4, 28029 Madrid, Spain

## Abstract

Changes in the uptake of many drugs by the target cells may dramatically affect the pharmacological response. Thus, downregulation of *SLC22A1*, which encodes the organic cation transporter type 1 (OCT1), may affect the response of healthy hepatocytes and liver cancer cells to cationic drugs, such as metformin and sorafenib, respectively. Moreover, the overall picture may be modified to a considerable extent by the preexistence or the appearance during the pathogenic process of genetic variants. Some rare OCT1 variants enhance transport activity, whereas other more frequent variants impair protein maturation, plasma membrane targeting or the function of this carrier, hence reducing intracellular active drug concentrations. Here, we review current knowledge of the role of OCT1 in modern liver pharmacology, which includes the use of cationic drugs to treat several diseases, some of them of great clinical relevance such as diabetes and primary liver cancer (cholangiocarcinoma and hepatocellular carcinoma). We conclude that modern pharmacology must consider the individual evaluation of OCT1 expression/function in the healthy liver and in the target tissue, particularly if this is a tumor, in order to predict the lack of response to cationic drugs and to be able to design individualized pharmacological treatments with the highest chances of success.

## 1. What Is the Interest of OCT1 in Modern Liver Pharmacology? 

Among the most important causes of cancer-related death worldwide are primary liver cancers, mainly hepatocellular carcinoma (HCC) and cholangiocarcinoma (CGC). These tumors can be resected surgically in only a limited number of cases because they are often in an advanced stage at the time of diagnosis, when surgery is no longer the recommended approach. Unfortunately, the efficacy of alternative treatments, including chemotherapy, is very poor in advanced liver cancer. Indeed, HCC and CGC are among the tumors with the highest drug refractoriness.

**Figure 1:**
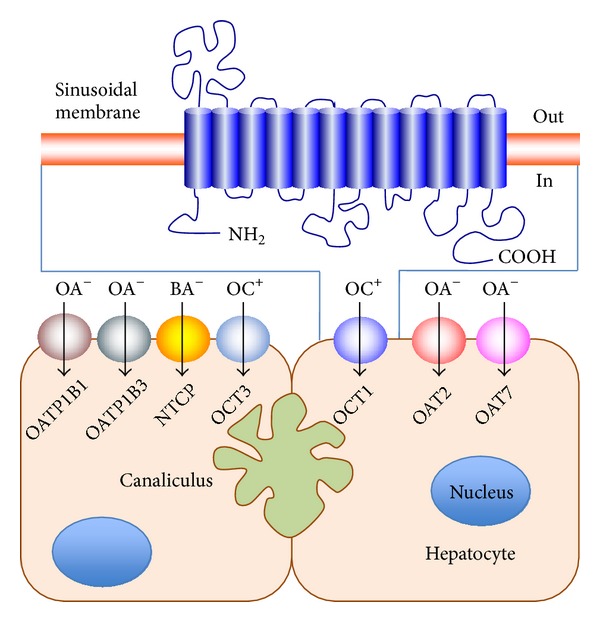
Schematic representation of carriers playing a major role in drug uptake across the sinusoidal membrane of human hepatocytes. The molecular model of the presumed OCT1 structure is also depicted. OA^−^: organic anions; OC^+^: organic cations; BA^−^: bile acids.

One of the most promising strategies to overcome liver cancer chemoresistance has been the use of tyrosine kinase inhibitors (TKIs), such as sorafenib. Despite its beneficial effect regarding the inhibition of tumor progression, the enhancement of overall survival is rather modest [1]. With respect to other TKIs, the mechanism of action of sorafenib depends on its access to intracellular targets. The organic cation transporter-1 (OCT1, gene symbol *SLC22A1*), located at the basolateral membrane of healthy hepatocytes (Figure 1), is involved in the uptake by the hepatocytes of xenobiotic (Table 1) and endogenous (Table 2) compounds. Regarding compounds with pharmacological interest, OCT1 is able to transport a large number of drugs (Table 3). Indeed, OCT1 is one of the transporters involved in sorafenib uptake. We have recently demonstrated that a marked decrease in OCT1 expression [2] and in the activity of this transporter due to the appearance of aberrant *SLC22A1* variants [3] may affect the response of HCC and CGC to sorafenib.

The interest of OCT1 in liver pharmacology is not limited to the recent use of TKIs in liver oncology [4], because OCT1 also mediates the uptake of other important cationic drugs (Table 3) such as metformin [5], platinum derivatives [6], and anthracyclines [7]. Therefore, the response to these drugs depends in part on OCT1 expression and function.

## 2. The *SLC22A* Family

Many endogenous or exogenous organic compounds handled by the liver are positively charged at physiological pH. Although they can have several different positively charged functional groups, the presence of tertiary or quaternary amine groups is very common. Quaternary amines are permanently charged, regardless of the pH of the medium, whereas the protonation state of tertiary amines depends on their pKa value and the pH of the medium. Since many of these compounds are highly hydrophilic, they cannot cross plasma membranes by simple diffusion. Accordingly, their uptake requires the involvement of plasma membrane transport systems, such as OCT1.

Based on their structural characteristics, organic cations have been classified as type I and type II categories [8]. The former includes small highly hydrophilic cations, usually below 500 Da [9]. Several quaternary ammonium compounds, such as tetraethylammonium (TEA) bromide and 1-methyl-4-phenylpyridinium (MPP^+^), are considered typical type I cations. Type II organic cations are less hydrophilic, bulky, and frequently polyvalent compounds, with *d*-tubocurarine and quinine being two of the typical members of this category [10].

The main transporters with the ability to translocate organic cations across the plasma membrane belong to the solute carrier family 22A (general gene symbol *SLC22A*) [11]. The human *SLC22A* family includes 13 well-characterized plasma membrane proteins: 3 organic cation transporters (OCTs), 3 Na^+^-zwitterion/cation cotransporters (OCTNs), and a heterogeneous group of transporters able to transport organic anions (OATs) or urate (URAT) [12]. Several members of this family are involved in the uptake of cationic (OCT) and anionic (OAT) drugs across the sinusoidal membrane of hepatocytes. An important role in the transport of organic anions across this membrane is also played by members of the SLCO (OATP1B1 and OATP1B3) and SLC10A (NTCP) families of carrier proteins (Figure 1).

## 3. Structure of the Organic Cation Transporter OCT1

Rat Oct1 was the first organic cation transporter to be cloned [13]. Later, its orthologs were cloned both in humans [14] and mice [15]. The human gene *SLC22A1* encoding OCT1 is localized within a cluster on chromosome 6q26 [16] and comprises 11 exons and 10 introns [17, 18]. The protein contains 554 amino acids with a predicted membrane topology similar to that of most members of the *SLC22A* family; that is, it comprises 12-*α*-helical transmembrane domains (TMD) with N- and C-termini localized intracellularly (Figure 1). There is a large extracellular loop between TMD1 and TMD2, containing glycosylation residues, and a large intracellular loop between TMD6 and TMD7, containing phosphorylation sites [19]. 

OCTs have several highly conserved sequence motifs compared to other members of the major facilitator superfamily, localized between TMD2 and TMD3 and between TMD8 and TMD9. Moreover, an 11-residue sequence found before TMD2 is considered a signature sequence of the OCT family [19]. Moreover, certain cysteine, glycine, and proline residues are conserved in all OCTs cloned to date, suggesting a key role for these residues in establishing the secondary structure of these proteins [20]. Indeed, OCTs show a marked homology between the N- and C-terminal halves, supporting the hypothesis that the structure of these proteins reflects a gene duplication event in the past [21]. Based on results from site-directed mutagenesis studies and models of protein tertiary structure prediction of rat Oct1, some amino acids have been suggested to be involved in the substrate translocation pore or in determining the affinity/selectivity for typical cations [22, 23]. 

## 4. Genetic Variants

It has been well established that the presence of genetic variants in genes encoding proteins involved in drug detoxification processes accounts for interindividual variability in drug response, and sometimes, this has severe consequences as regards drug toxicity and therapeutic efficacy [24]. Genetic polymorphisms in genes encoding drug transporters have also been suggested as a possible mechanism accounting for interindividual variability in drug response by altering pharmacokinetic and hepatic drug clearance [25]. Because a broad variety of drugs used in clinical practice are organic cations, the existence of genetic variants in the *SLC22A1* gene has relevant clinical implications in human pharmacology. Thus, more than 1000 mutations in the *SLC22A1* gene, in the promoter region, in the coding sequence, in the 5′UTR and 3′UTR-regions, or in the introns have been described. However, the biological significance of most single-nucleotide polymorphisms (SNPs) in noncoding regions remains to be elucidated [26–28]. Moreover, the expression of truncated OCT1 isoforms originated by alternative splicing mechanisms, such as exon skipping and intron retention, has also been found predominantly in tumor cells [3, 18]. These OCT1 variants resulting in truncated proteins have been reported to be nonfunctional [18]. For example, the c.1276+1insGTAAGTTG variant, which consists of an 8-bp insertion of intron 7 between exons 7 and 8, results in a truncated protein that has recently been associated with adverse side effects in patients treated with metformin [29].

Regarding the coding sequence of OCT1, the described modifications deposited in the NCBI database include one 3-bp deletion (M420del), 8 nonsense mutations, and 49 missense mutations. Our group has recently identified the existence of 3 additional OCT1 SNPs in HCC and CGC [3]. 

Several common nonsynonymous mutations have been found in the *SLC22A1* gene in individuals from many ethnic groups, and some of these mutations, such as L160F, P341L, and M408V, have been identified in all of them [30]. These variants, which appear with relatively high frequency, have been reported to maintain transport ability [5]. However, it has also been reported that patients with chronic myeloid leukemia bearing the wild-type genotype GG of the L160F variant show a poorer response to imatinib than patients with the mutation [31].

Some of the SNPs that result in amino acid substitution severely reduce and alter substrate transport as measured in cellular assays, or may even be of important clinical relevance [26, 28, 30, 32–35]. Thus, in *in vitro* assays carried out using metformin, MPP^+^, or TEA as prototypical substrates, a reduced or even abolished OCT1-mediated transport activity was observed for R61C, C88R, S189L, G220V, P341L, G401S, M420del, G465R, P283L, R287G, P117L, Q97K, R206C, R61S fs∗10, and C88A fs∗16 (Table 4). It is striking that several of the variants with reduced activity had altered evolutionarily conserved glycine residues; that is, G220V, G401S, and G465R, suggesting that these residues may be particularly important for OCT1 function [30, 36].

Amino acid variants with increased activity are rare, but they are of special biological interest because they provide information about the residues governing protein activity and perhaps substrate specificity. Several studies have demonstrated that the S14F variant has an increased MPP^+^ transport activity [30]. However, the functional consequences of the presence of *SLC22A1* genetic variants may differ, depending on the OCT1 substrate. Thus, the S14F variant has an impaired ability to take up metformin [36], whereas TEA transport is not affected [3]. The P283L and P341L variants do not affect metformin uptake, but do reduce that of MPP^+^ [26, 30]. Changes in substrate selectivity for the C88R and G401S variants have also been described [26].

The expression of OCT1 variants with reduced activity may lead to the accumulation of toxic metabolites and enhanced exposure to environmental toxins, such as the piperidine derivative 1-methyl-4-phenyl-1,2,3,6-tetrahydropyridine (MPTP), which may reach the brain and be involved in the development of neurodegenerative diseases [37, 38].

OCT1 variants may contribute to reducing therapeutic drug responses, presumably by decreasing the hepatic uptake of these drugs. Examples are metformin [5, 29, 36, 39], sorafenib [3], levodopa [40], lamivudine [41], some platinum analogs [42], tropisetron and ondansetron [43]. Furthermore, dramatic differences in the liver distribution of metaiodobenzylguanidine and metformin have been observed in Oct1-knockout mice as compared with wild-type mice [44, 45].

As previously mentioned, a significant association of *SLC22A1* mRNA transcription levels with the success of imatinib-based therapy in leukemia patients has been reported [46–48]. However, the presence of genetic variants may markedly change the situation [31]. For instance, M420del may alter imatinib pharmacodynamics without changing its systemic pharmacokinetics, resulting in a reduction in the efficacy of this drug in the specific target leukemic cells [49]. Similar results have recently been reported for the use of sorafenib in the treatment of liver cancer [3]. Confocal microscopy studies have revealed that in some cases, the reason accounting for the absence of transport function in OCT1 variants is an aberrant targeting of the protein to the plasma membrane (Table 4), whereas variants that maintain transport ability unaltered or diminished are always localized at the plasma membrane [3, 30, 35, 36].

## 5. Tissue Distribution 

In humans, OCT1 has a broad tissue distribution, although it is primarily expressed in hepatocytes [50]. In rat hepatocytes, Oct1 has been located at the sinusoidal membrane [51]. OCT1 is also expressed in cholangiocytes [34, 52]. Although to a lesser extent than in rodent kidney, human OCT1 is also expressed at the apical membrane of epithelial cells in the proximal and distal tubules of the nephron [7]. OCT1 is also expressed at the basolateral membrane of enterocytes, where, together with the combined transport activity of carriers localized at the apical membrane of these cells, it accounts for the secretion of organic cations toward the intestinal lumen [7]. This tissue distribution differs from that of other OCTs. Thus, human OCT2 is mostly expressed in the kidney, but not in liver [50], whereas human OCT3 has a widespread tissue distribution, with the highest expression in liver and placenta [53].

## 6. Functional Characteristics

The sensitivity of rat Oct1 [13] and human OCT1 [50, 54] the electrical potential across the plasma membrane suggests that these, as well as other OCT isoforms, translocate organic cations in an electrogenic manner that is not dependent on Na^+^ or H^+^ gradients [50]. OCT-mediated transport may occur across the plasma membrane in both directions [7]. OCTs share broad substrate specificity since they can translocate a wide variety of structurally unrelated compounds. Most OCT substrates are organic cations or weak bases that are positively charged at physiological pH, although uncharged compounds may also be transported. Together, OCT substrates include endogenous compounds and clinically important drugs and toxins. 

Owing to its localization and substrate specificity, the main physiological role of OCT1 is the detoxification of endogenous cationic compounds, but it is also involved in drug disposition [44]. In hepatocytes, OCT1 accounts for the first step in the detoxification of endogenous and xenobiotic compounds, including many drugs, since it carries out uptake across the sinusoidal membrane. Because OCT1 can mediate not only the uptake but also the extrusion of substrates, this transporter may be also involved in the release of organic cations from hepatocytes to blood [7].

## 7. OCT1 Substrates

Regarding chemical structure, model OCT1 substrates include type I cations such as tetraalkylammonium compounds-TEA [14], tetramethylammonium [50], tetrapropylammonium [55], tetrabutylammonium [55], tributylmethylammonium [10], *N* ^1^-methylnicotinamide, MPP^+^ [50], azidoprocainamide methoiodide [10], and 4-[4-(dimethylamino)-styryl]-N-methylpyridinium (ASP) [56, 57]. Some type II cations such as N-methyl-quinine and N-methyl-quinidine are also transported by OCT1 [10] (Table 1). 

Regarding the biological characteristics of the xenobiotic substrates of OCT1, this transporter is involved in the uptake of several toxins (Table 1) as well as endogenous compounds (Table 2). OCT1 appears to govern the hepatic uptake and elimination of MPTP, which is a toxic metabolite involved in the development of Parkinsonian syndrome [37, 38]. Thus, an impairment in OCT1 expression may lead to enhanced exposure to MPTP and contribute to the development of neurodegenerative disease [30].

Several guanidine compounds, described as uremic toxins, are accumulated in the blood of patients with renal insufficiency [73, 74]. In humans, OCT1 and OCT2 are involved in the uptake of uremic guanidine compounds such as guanidinosuccinic acid, methylguanidine and guanidinovaleric acid [65].

Fluorescent dyes, such as ethidium bromide, which is routinely used in cell viability determination assays, are transported by the three human OCT isoforms [63]. Moreover, OCT1 is also able to transport 4′,6-diamidino-2-phenylindole (DAPI), another fluorescent compound widely used to stain cell nuclei. Other toxic OCT1 substrates are nicotine [59] and herbicides, such as paraquat [64].

Neurotransmitters with an amine structure, such as catecholamines (dopamine, epinephrine, and norepinephrine), serotonin, acetylcholine, and histamine are also transported by OCT1 (Table 2), which is involved in the synaptic recycling of most of these neurotransmitters [44].

OCT1 may also play a role in the transport of prostaglandins, such as E2 and F2a [70]. This is a matter of controversy, because at physiological pH, prostaglandins are negatively charged [75]. In addition, some steroid hormones, such as progesterone, *β*-estradiol, and corticosterone, behave as inhibitors of human OCT1 [76].

The polyamines putrescine and spermidine can be transported by human OCT1 (and also by human OCT2 and OCT3) [77]. Owing to the fact that these compounds play prominent roles in cell growth, proliferation, and differentiation, control of the cytosolic polyamine pool is critical for normal physiology. Indeed, alterations in polyamine homeostasis have been linked to stroke, renal failure, and cancer [78]. Other biogenic amines transported by human OCT1 are choline, creatinine, L-carnitine, guanidine, and the L-arginine metabolite agmatine.

In addition, OCTs are sensitive to inhibition by compounds that are not transported [7]. Type II cations inhibit OCT1-mediated uptake of type I cations, but they are not transported themselves [10]. Type II includes a variety of cations, such as tetrapentylammonium [50, 79], decynium 22 [76], and disprocynium 24 [80]. The OCT1 inhibitors with the highest affinity are atropine [81] and prazosin [76]. 

## 8. Regulation of OCT1 Expression and Function

The regulatory mechanisms of OCT1 expression and function are important because they can alter the disposition of the large list of endogenous substrates or drugs commented previously (Tables 1–4).

Short-term regulation by post-translational mechanisms may result in changes in protein trafficking toward the plasma membrane or in transport affinity, which may occur in response to specific stimuli able to activate phosphorylation or dephosphorylation processes [82]. The results from experiments carried out in two different expression systems—Chinese hamster ovary (CHO) cells and HEK293 cells—have suggested that OCT1 is activated by the Src-like p56^lck^ tyrosine kinase [83]. The Ca^2+^/calmodulin pathway also stimulates the post-transcriptional regulation of OCT1 [84]. Moreover, this study revealed that calmodulin-dependent protein kinase II (CaMKII), a downstream component of this pathway, is involved in OCT1 regulation. The activation of protein kinase C (PKC) decreases the affinity of OCT1 for prototypical substrates [56, 85].

Regarding long-term regulation, the human OCT1 promoter contains two adjacent putative DNA-response elements for the hepatocyte nuclear factor-4*α* (HNF-4*α*) [86]. The interaction of HNF-4*α* with these response elements activates OCT1 transcription, which can be inhibited through the small heterodimer partner (SHP). Since the expression of this transcriptional corepressor can be induced by bile acids [86], OCT1 expression is reduced in cholestatic liver disease, when elevated bile acid levels counterbalance the HNF-4*α*-mediated activation of OCT1 transcription [34].

## 9. OCT1 Expression in the Liver under Pathological Conditions

Genetic and epigenetic factors account for the marked interindividual variability in OCT1 expression levels in healthy human livers [34]. In addition, pathological conditions, such as cholestasis [34] and drug exposure, may further modify OCT1 expression. We have recently described that primary liver cancer derived from epithelial cells, that is, HCC, CGC, and hepatoblastoma, shares a decreased expression of OCT1 as a common feature [2]. The reduction in the expression levels of this transporter has been associated with advanced tumor stages and poorer overall patient survival rates in HCC [87] and CGC [52]. Hypermethylation of the *SLC22A1* promoter has been suggested to be the mechanism accounting for the downregulation of OCT1 in HCC [77].

## 10. Role of OCT1 in Drug Transport

The high expression level of OCT1 at the sinusoidal membrane of hepatocytes accounts for the relevance of this transporter in the handling of many cationic drugs by the liver (Table 3). The role of OCT1 in the pharmacokinetics or even the pharmacodynamic of these drugs depends on several factors, such as the step in which the transporter is involved (uptake or efflux), the type of drug transported (e.g., antineoplastic), and the existence of interactions among OCT1 substrates.

- * Role of OCT1 in the Uptake of Drugs Targeted to Hepatocytes.* An important example of this type of drug is metformin, a hydrophilic organic cation with a biguanide structure that is widely used for the treatment of type 2 diabetes. OCT1 is responsible for the uptake of metformin by the liver [5]. In this organ, metformin exerts its main pharmacological activity by decreasing gluconeogenesis, which results in a reduction in blood glucose levels. Accordingly, the variability in OCT1 expression, and hence, in the ability of the liver to take up metformin, may play an important role in the inter-individual difference regarding the clinical efficacy of this drug [103]. Another example of this type of drug is lamivudine, a cytidine analog that, through OCT1, is efficiently taken up by hepatocytes, where its active metabolites prevent hepatitis B virus replication [101].
- * Role of OCT1 in Drug Efflux from Hepatocytes to Blood*. Acyclovir and ganciclovir are two OCT1 substrates with antiviral activity [102]. Owing to their poor intestinal absorption, they are administered as l-valyl esters, that is, valaciclovir and valganciclovir, respectively. These prodrugs undergo hepatic first-pass metabolism, being converted by hepatocyte esterases into their active forms. Since OCT1 can mediate both the uptake and efflux of its substrates [7], this transporter is also involved in the release of acyclovir and ganciclovir towards the blood, from where they can reach their targets.

O-Desmethyltramadol is an opioid analgesic and the main active metabolite of tramadol, produced in the liver by demethylation by the cytochrome P450 (CYP) enzyme CYP2D6. O-Desmethyltramadol, but not tramadol, is an OCT1 substrate [88]. The interindividual differences observed in the OCT1 expression and/or activity affect O-desmethyltramadol pharmacokinetics and thus the efficacy of tramadol treatment [88].

(iii) * Role of OCT1 in the Detoxification of Cationic Drugs*. Many drugs undergo a first-pass detoxification through the liver that may dramatically reduce systemic exposure to them and limit their pharmacological activities. The important role of the liver in detoxification processes involves a series of complex events that include uptake across the sinusoidal membrane of hepatocytes (phase 0), intracellular biotransformation (phases I and II), and extrusion across the canalicular membrane into bile (phase IIIa) or, alternatively, across the sinusoidal membrane back into the blood (phase IIIb). As previously mentioned, OCT1, together with other members of the *SLC22A* family of transporters, can play an important role in the clearance of most cationic drugs from hepatic sinusoidal blood [7]. OCT1-mediated renal clearance and a reduction in the drug intestinal absorption also contribute to a reduction in drug bioavailability [7]. Several examples serve to illustrate the role of OCT1 in drug detoxification.

The major detoxification route of the serotonin receptor type antagonists, ondansetron and tropisetron, used to treat chemotherapy-induced nausea and vomiting, consists of OCT1-mediated liver uptake and subsequent metabolic inactivation by CYP enzymes, mainly CYP2D6 [43]. The level of OCT1 expression in the liver therefore determines the pharmacokinetics and efficacy of both drugs [43].

Similarly, OCT1 plays a key role in the first-pass effect through the liver and hence in the bioavailability of other cationic drugs, such as amantadine, levodopa and pramipexole [40], cimetidine [91], ciprofloxacin and other fluoroquinolones [92, 93], furamidine and pentamidine [100], lamotrigine [89], sulpiride [90], and zalcitabine [101]. 

Most type II organic cations, typically hydrophobic, bulky, and polyvalent, such as atropine, decynium-22, prazosin, quinine, and *d*-tubocurarine, are able to inhibit OCT1, but are not translocated [7, 10]. Some exceptions of clinically used type II organic cations transported by OCT1 are quinidine, pancuronium, and rocuronium [10].

(iv) * Role of OCT1 in the Uptake of Antitumor Drugs*. OCT1 may also be critical for therapeutic concentrations of certain cationic antineoplastic drugs, such as mitoxantrone, to be reached in tumor cells [7]. Similarly, intracellular concentrations of m-iodobenzylguanidine radiolabeled with iodine-123 (for imaging purposes) or with iodine-131 (a therapeutic radiopharmaceutical) [96] are dependent on OCT1 expression/function. Another antineoplastic drug taken up by cancer cells through OCT1 is sepantronium bromide or YM 155, a novel survivin suppressant that exhibits potent antitumor activity against solid human tumors and lymphoma cells [97]. Bleomycin, an antibiotic with anticancer activity, is transported in yeasts by a polyamine transport system [103] whose human ortholog is probably OCT1 [104]. In a recent study, the authors suggest that OCT1 can contribute to the cellular uptake of irinotecan, paclitaxel, and mitoxantrone by cancer cells [94]. It was shown that OCT1-expressing cell lines derived from lymphoma and OCT1-transfected CHO cells exhibited higher sensitivity to the cytotoxic effects of irinotecan and paclitaxel than cells that did not express OCT1 [94]. Moreover, both drugs, as well as mitoxantrone, were able to inhibit the uptake of a typical OCT1 substrate by OCT1-transfected cells [94].

Some platinum derivatives are also transported by OCT1. Thus, the intracellular accumulation, and hence, toxicity, of oxaliplatin and picoplatin, but not that of cisplatin and carboplatin, are markedly increased in OCT1-transfected cells, suggesting that oxaliplatin [6] and picoplatin [105] could be good substrates for this transporter. With a view to obtain platinum derivatives that can be recognized by carrier proteins able to transport cholephilic compounds, and hence to enhance their targeting toward the hepatobiliary system, our group synthesized a new family of antitumor compounds by binding cisplatin to bile acids. Among these, cis-diammine-chloro-cholylglycinate-platinum (II) or Bamet-R2 and cis-diammine-bisursodeoxycholate-platinum (II) or Bamet-UD2 showed liver organotropism, strong *in vitro* cytostatic activity, and an antitumor effect against tumor xenografts implanted in the livers of athymic *nu/nu* (nude) mice [106, 107]. Bamets are neutral compounds that act as substrates of several members of transporters belonging to the *SLC10A* and *SLCO* families [95]. In aqueous solution, Bamets undergo replacement of some of the platinum (II) ligands by water, resulting in DNA-reactive cationic derivatives that are transported by OCT1 [95]. 

TKIs constitute a novel therapeutic strategy designed with the aim of interacting with the molecular targets involved in the apoptosis/survival pathways of tumor cells. These drugs are effective in the treatment of several types of cancer [108]. Among other targets, imatinib is a potent inhibitor of BCR-ABL tyrosine kinase. This drug has mainly been used in the treatment of chronic myeloid leukemia, where it displays substantial efficacy regarding prolonged survival and improved quality of life [108]. In chronic myeloid leukemia primary cells and cell lines, imatinib uptake has been shown to be dependent on OCT1 [98]. Experiments carried out with OCT1-transfected cells support this hypothesis [4]. The affinity constant of human OCT1 for imatinib has been calculated to be approximately 5 *μ*M [99]. The degree of OCT1 expression has been suggested to be a useful biomarker to predict the success of imatinib-based therapy in leukemia patients, and, furthermore, leukemia patients who had higher OCT1 expression levels showed a better response to the drug [47, 48].

There is no efficient standard therapy to treat patients with advanced liver cancer. Regarding chemotherapy, most drugs assayed have shown no relevant antitumor effect or survival benefit. Erlotinib and gefitinib are TKIs, OCT1 substrates [4], that have been evaluated in patients with advanced HCC. In a phase-II study in patients with advanced HCC, erlotinib showed an admissible tolerance profile and limited clinical activity, as revealed by disease control [109]. Gefitinib has been shown to inhibit cell proliferation and metastatic spread in HCC cell lines and in *in vivo* mice models, respectively, although its clinical efficacy is low [110].

Sorafenib is a new effective chemotherapeutic agent that has been approved for the treatment of HCC patients and is the only one shown to improve overall survival benefit in patients with the disease [1]. Sorafenib is an inhibitor of several tyrosine protein kinases, such as VEGFR, PDGFR, and Raf kinases, blocking the molecular pathways involved in tumor progression and angiogenesis [111]. In recent studies carried out by our group, we have demonstrated that OCT1 expression in oocytes of the frog *Xenopus laevis* induces the ability to take up sorafenib in these cells in a quinine-sensitive manner [3]. Moreover, transfection of OCT1 to cells derived from liver cancer was able to confer them higher sensitivity to the toxic effect of sorafenib [3]. Studies carried out in human hepatocytes and OCT1-transfected CHO cells have suggested that in addition to OCT1, other transporters may also be involved in sorafenib uptake by human hepatocytes [112].

(v) * Role of Interactions among OCT1 Substrates in Liver Pharmacology.* Certain endogenous substances and food components may be involved in interactions with OCT1, and hence impair the bioavailability of the cationic drugs previously commented. The existence of OCT1 substrates able to affect the bioavailability of cationic drugs in patients' blood may also account for the interindividual variability in the pharmacological activity of several other drugs [113]. Moreover, since a wide variety of clinically used drugs are organic cations, drug/drug interactions between OCT1 substrates and/or inhibitors are highly likely. For example, the OCT1 substrates tropisetron and ondansetron are two antiemetics usually administered together with some antitumor drugs to prevent chemotherapy-induced nausea and vomiting. In the case of treatments based on cationic antineoplastic drugs, the combined treatment may reduce the uptake of these drugs by the tumor and hence decrease the efficacy of the chemotherapy [43]. Additional examples for such a clinically relevant interaction include antibiotics and antihypertensives [92].

## 11. Perspectives

The relevant role of OCT1 in human liver pharmacology accounts for the importance of the observed changes in its expression levels, as well as the presence of nonfunctional genetic variants in the overall response to treatments based on the administration of cationic drugs that are substrates of this carrier. Thus, modern pharmacology must consider the possibility of evaluating the OCT1 profile regarding expression/function in the healthy liver and in the target tissue, particularly if this is a tumor, of each patient in order to predict the lack of response and to design an individualized pharmacological regimen with the highest likelihood of success. 

## Figures and Tables

**Table 1:** Xenobiotic compounds transported by human OCT1.

Category	Compounds	References
Alkaloids	APD-ajmalinium	[10]
	Berberine	[58]
	Nicotine	[59]

Fluorescent dyes	4′,6-diamidino-2-phenylindole (DAPI)	[60]
	4-(4-(dimethylamino)-styryl)-N-methylpyridinium (ASP)	[56, 61]
	[2-(4-nitro-2,1,3-benzoxadiazole-7-yl)aminoethyl]trimethylammonium	[62]
	Ethidium	[63]

Herbicides	Paraquat	[64]

Neurotoxins	1-Methyl-4-phenylpyridinium (MPP^+^)	[50]
	1-Methyl-4-phenyl-tetrahydropyridine (MPTP)	[38]

Quaternary ammonium compounds	Tetraalkylammonium	[10]

Uremic toxins	Guanidinosuccinic acid	[65]
	Methylguanidine	
	Guanidinovaleric acid	

Mycotoxins	Aflatoxin B1	[66]

**Table 2:** Endogenous compounds transported by human OCT1.

Category	Compounds	References
Catecholamines	Dopamine	[67]
	Epinephrine	
	Norepinephrine	
Ethanolamines	Choline	[11]
Biogenic monoamines	Histamine	[67]
	Serotonin	
Biogenic polyamines	Spermidine	[68]
	Putrescine	
	Agmatine	[69]
Prostaglandins	Prostaglandin E2	[70]
	Prostaglandin F2*α*	
Vitamins	Thiamine	[71]
	*N* ^ 1^-Methylnicotinamide	[50]
Other metabolites	Creatinine	[72]
	L-carnitine	

**Table 3:** Drugs transported by human OCT1.

Drug category	Typical compounds	Pharmacological action	Reference
Opioids	O-Desmethyltramadol	Analgesic	[88]
*Cinchona* alkaloids	Quinidine	Antiarrhythmic	[10]
Calcium channel blockers	Lamotrigine	Anticonvulsant	[89]
Dopamine antagonists	Sulpiride	Antidepressive	[90]
Serotonin antagonists	Ondansetron, tropisetron	Antiemetic	[43]
Histamine antagonists	Cimetidine	Antigastric ulcers	[91]
Fluoroquinolones	Ciprofloxacin	Antimicrobial	[92, 93]
Anthraquinones	Mitoxantrone	Antineoplastic	[7]
Camptothecin analogs	Irinotecan	Antineoplastic	[94]
Glycopeptide antibiotics	Bleomycin	Antineoplastic	[7]
Platinum compounds	Oxaliplatin, Bamet-UD2	Antineoplastic	[6, 95]
Radiopharmaceuticals	[^123^I] or [^131^I] m-iodobenzylguanidine	Antineoplastic	[96]
Survivin suppressants	YM 155	Antineoplastic	[97]
Taxoids	Paclitaxel	Antineoplastic	[94]
Tyrosine kinase inhibitors	Imatinib	Antineoplastic	[3, 98, 99]
	Sorafenib		
Aromatic diamidines	Furamidine, pentamidine	Antiparasitic	[100]
NMDA receptor antagonists	Amantadine	Anti-Parkinsonian	[40]
Dopamine agonists	Levodopa, pramipexole	Anti-Parkinsonian	[40]
Nucleoside analogs	Acyclovir, lamivudine	Antivirals	[101, 102]
Biguanides	Metformin	Hypoglycemic	[5]
Cationic steroids	Pancuronium Rocuronium	Neuromuscular blocking	[10]

**Table 4:** Genetic variants and functional consequences in human OCT1.

Reference SNP	Amino acid change	Nucleotide variation	Exon	Protein domain	Transport activity compared with wild type				PML
					MPP^*+*^	TEA	Metformin	Others	
rs34447885	S14F	c.41C>T	1	NH2 terminus	↑ [30]	= [3]	↓ [36]	= Sorafenib [3]	
rs12208357	R61C	c.181C>T	1	Large EL	↓ [26, 30]	↓ [3, 26]	↓ [36]	↓ Sorafenib [3]	NO [36]
NA	R61S fs∗10	c.181delCGinsT	1	Large EL		↓ [3]		↓ Sorafenib [3]	
rs35546288	L85F	c.253C>G	1	Large El	= [30]				NO [3]
rs55918055	C88R	c.262T>C	1	Large EL	↓ [26]	↓ [3]		↓ Sorafenib [3]	NO [3]
NA	C88A fs∗16	c.262delT	1	Large EL		↓ [3]		↓ Sorafenib [3]	NO [3]
NA	Q97K	c.289C>A	1	Large EL			↓ [35]		YES [35]
NA	P117L	c.350C>T	1	Large EL			↓ [35]		YES [35]
rs683369	L160F	c.480G>C	2	TMD2	= [26, 30, 33]	= [3, 33]	= [36]	= Sorafenib [3]	YES [33]
rs34104736	S189L	c.566C>T	3	TMD3	= [30]	↓ [3]	↓ [36]	↓ Sorafenib [3]	NO [3]
NA	P197S	c.589C>T	3	TMD3		= [3]		= Sorafenib [3]	YES [3]
NA	R206C	c.616C>T	3	EL2			↓ [35]		NO [35]
rs36103319	G220V	c.659G>T	3	TMD4	↓ [30]	↓ [3]	↓ [36]	= Sorafenib [3]	
rs4646277	P283L	c.848C>T	5	TMD6	↓[33, 41]	↓ [32, 33, 41]	= [41]	↓ Lamivudine [41]	YES [33]
rs4646278	R287G	c.859C>G	5	Large CL	↓ [33]	↓ [3, 32, 33]		↓ Sorafenib [3]	YES [33]
rs2282143	P341L	c.1022C>T	6	Large CL	↓ [30, 33, 41]	↓ [32, 33, 41]	= [36, 41]	↓ Lamivudine [41]	YES [33]
rs34205214	R342H	c.1025G>A	6	Large CL	= [30]		= [36]		
rs34130495	G401S	c.1201G>A	7	CL4	↓ [26, 30]	↓ [26] = [3]	↓ [36]	= Sorafenib [3]	
rs628031	M408V	c.1222A>G	7	TMD9	= [30]		= [36]		
rs20222080	M420del	c.1258_1260delATG	7	TMD9	= [26, 30]		↓ [36]		
rs35956182	M440I	c.1320G>A	7	TMD10	= [30]				
rs34295611	V461I	c.1381G>A	7	CL5	= [30]				
rs34059508	G465R	c.1393G>C	8	TMD11	↓ [30]	↓ [3]	↓ [36]	= Sorafenib [3]	YES [3]
rs35270274	R488M	c.1463G>C	8	EL6	= [30]		= [36]		YES [30]

EL: extracellular loop; CL: cytoplasmic loop; NA: not assigned; PML: plasma membrane localization; TMD: transmembrane domain; ↑: increased; ↓: decreased; =: unaltered.

## Abbreviations

CGC: cholangiocarcinoma
HCC: hepatocellular carcinoma
MOC: mechanism of chemoresistance
MPP^+^: 1-methyl-4-phenylpyridinium
OCT: organic cation transporter
SNP: single nucleotide polymorphism
TEA: tetraethylammonium
TKI: tyrosine kinase inhibitor.
